# Supplementary material for: Kinetoplastid Species Maintained by a Small Mammal Community in the Pantanal Biome
Source: Pathogens. 2022 Oct 19;11(10):1205. doi: 10.3390/pathogens11101205 (PMC9612235; doi:10.3390/pathogens11101205)
Supplement: Supplementary file 1 [file pathogens-11-01205-s001.zip › pathogens-1961379-supplementary.pdf]

Table S1. Sequencing data quality for the analyzed samples expressed by: input — number of reads in raw fastq files; filtered — number of reads after preliminary quality filtering; denoised (F/R) — number of reads after quality filtering; merged — number of merged forward-reverse reads; nonchim — number of merged reads after removal of chimera sequences; % passed — relative number of passed reads after all the above steps.

**Host**	**Input**	**Filtered**	**Denoised F**	**Denoised R**	**Merged**	**Nonchim**	**% passed**
Monodelphis domestica	35704	27750	27657	27656	27594	27445	99.46%
Thylamys macrurus	73610	67151	66772	66936	65233	61250	93.89%
Oecomys mamorae	146617	123626	123201	123124	121734	120501	98.99%
Thrichomys fosteri	39032	28847	28758	28726	28585	28559	99.91%
Clyomys laticeps	70233	59959	59868	59873	59779	59468	99.48%
Holochilus chacaris	52097	47846	47779	47818	47662	47662	100.00%
